# Principles of Human Physiology

**Published:** 1856-01

**Authors:** 


*Principles of Human Physiology.* By William B. Carpenter, M. D. &c. A new American Edition, by F. Gurney Smith, M. D. Phila—Blanchard & Lea.

We feel that when we say that this edition is a carefully revised one by the eminent author, and that Dr. Smith has also added to it, we scarcely need add one word of comment; and indeed we shall say no more than that *it is the very best work extant on the subject of which it treats*, which we think will be sufficiently explicit and satisfactory. The publishers, in this instance, deserve to be noticed and commended, for the excellent and numerous illustrations which adorn its pages.

